# Supplementary material for: Upregulation of Wheat Heat Shock Transcription Factor TaHsfC3-4 by ABA Contributes to Drought Tolerance
Source: Int J Mol Sci. 2024 Jan 12;25(2):977. doi: 10.3390/ijms25020977 (PMC10816066; doi:10.3390/ijms25020977)
Supplement: Supplementary file 1 [file ijms-25-00977-s001.zip › ijms-2774464-supplementary.pdf]

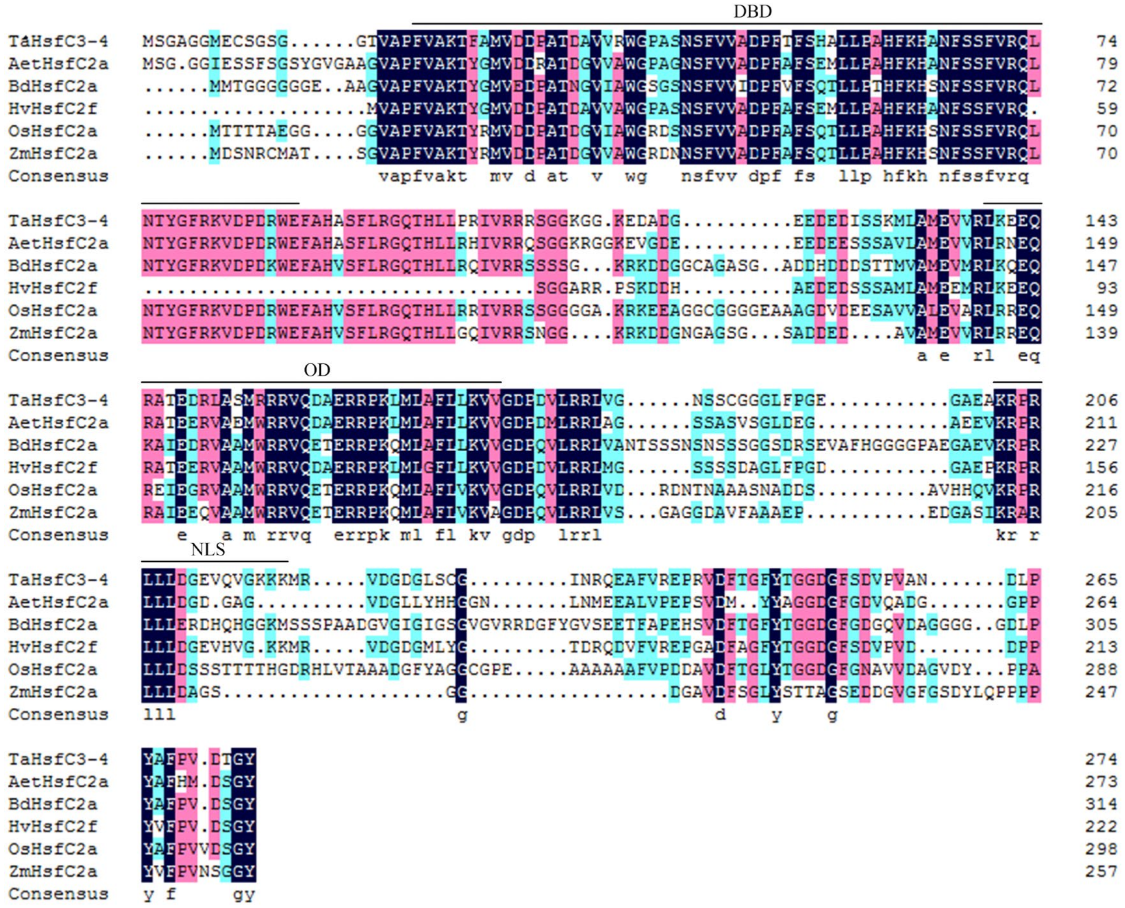

Figure S1. Sequence alignment of TaHsfC3-4 and HsfCs from other species. Multiple sequence alignment of amino acid sequences from *Triticum aestivum* (TaHsfC3-4), *Aegilops tauschii* (AetHsfC2a), *Brachypodium distachyon* (BdHsfC2a), *Hordeum vulgare* (HvHsfC2f), Oryza sativa (OsHsfC2a) and *Zea mays* (ZmHsfC2a). Amino acids shaded by color are conserved, with black indicating the highest similarity, red less, and blue least. Conservative domains are marked with line segments.

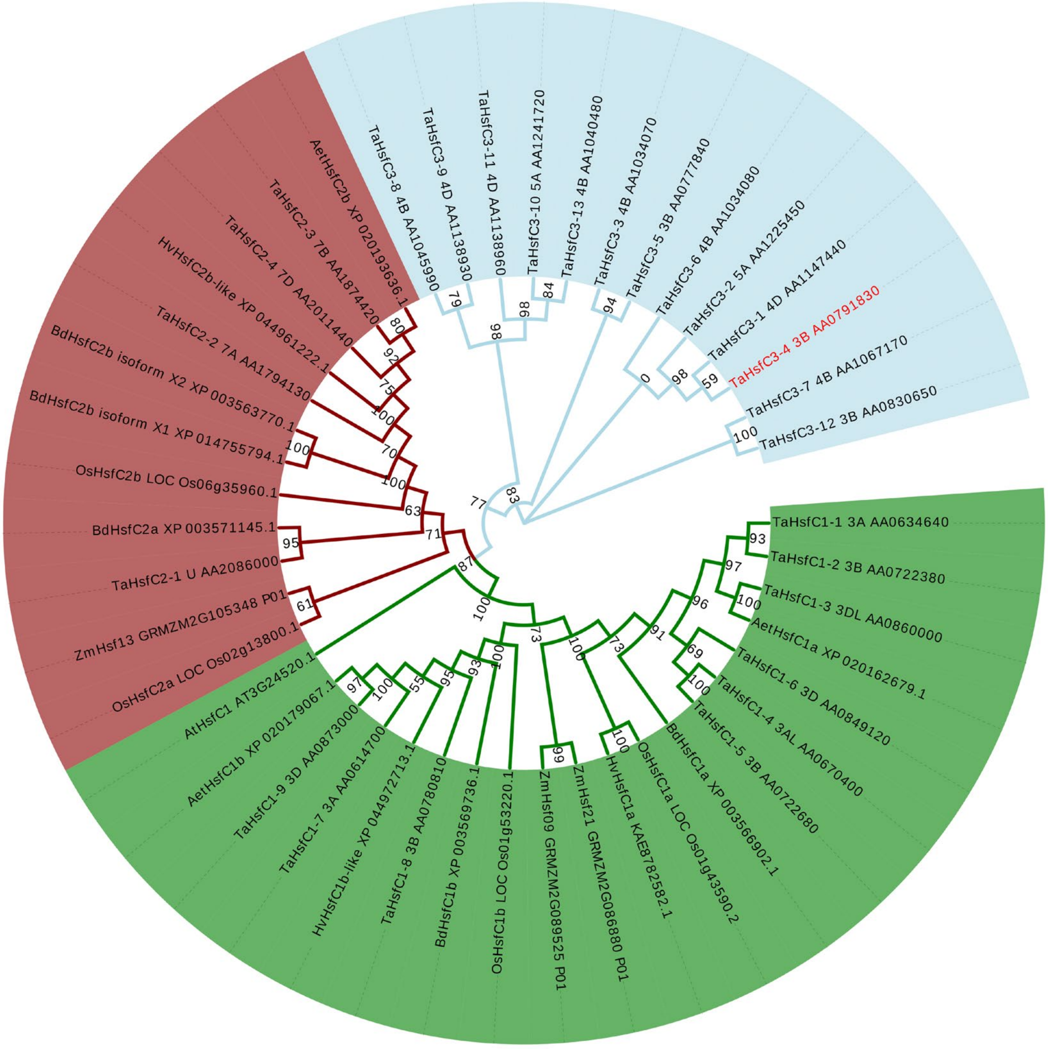

Figure S2. Phylogenetic tree of TaHsfC3-4 and HsfCs from other species.

The phylogenetic tree was constructed by the neighbor-joining method using MEGA X software. The percentage of replicate trees in which the associated taxa clustered together in the bootstrap test (1000 replicates) are shown next to the branches. The evolutionary distances were computed using the Poisson correction method. Subclass C1, C2 and C3 Hsfs were in green, darkred and lightblue, respectively. TaHsfC3-4 was marked with red characters.

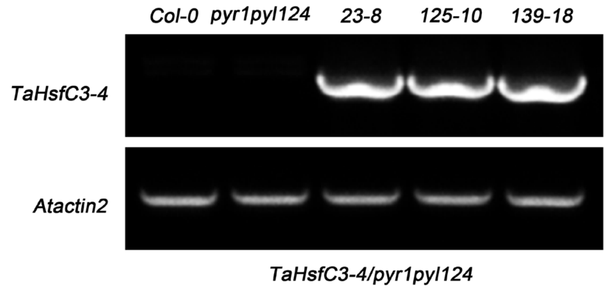

Figure S3. Identification of transgenic plants.

The mRNA levels of *TaHsfC3-4* overexpressing lines under *pyr1pyl124* genetic background, *pyr1pyl124* quadruple mutant and *Col-0* were examined by semi-quantitative RT-PCR analysis, and the transcript levels of *Atactin2* was used as a loading control [52].

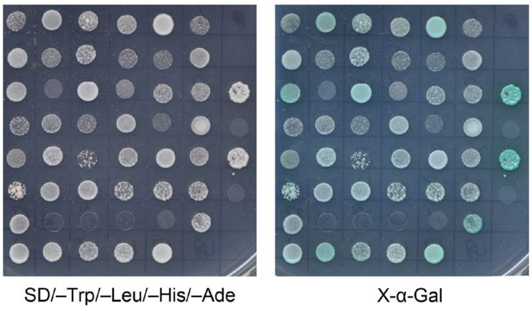

Figure S4. Screening of TaHsfC3-4-interacting proteins.

Normalized yeast cDNA library is constructed by using RNA extracted from leaves of drought-treated wheat variety C6005. During Y2H screening, TaHsfC3-4 was used as a bait to identify potential protein interaction partners. The interaction was evaluated by the yeast growth on the selective medium QDO (SD/–Trp/–Leu/–His/–Ade) with X-α-Gal.

Table S1 Primers used in this study

Assays	Gene	Forward primer	Reverse primer
RT-PCR	TaHsfC3-4	GCCCACTTCAAGCACGCCAACTTCTC	CTTCTTCTTGCCCACCTGGACCTCCC
	Actin2	CAATCGTGTGTGACAATGG	AACCCTCGTAGATTGGCA
RT-qPCR	TaHsfC3-4	ACGGGCTGTCCTGCGGCATCAA	CGCCTCCGGTGTAGAAACCAGTGAA
	TaRP15	GCACACGTGCTTTGCAGATAAG	GCCCTCAAGCTCAACCATAACT
Transactivation analysis	TaHsfC3-4	TCAGAGGAGGACCTGCATATGATGAGC GGCGCCGGCGGCATG	TCGACGGATCCCCGGGAATTCTCAGTA GCCGGTGTCCACAGGGA
Subcellular localization	TaHsfC3-4	GAGAACACGGGGGACTCTAGAATGAG CGGCGCCGGCGGCATG	GCCCTTGCTCACCATGGATCCGTAGCC GGTGTCCACAGGGA
Genetic transformation	TaHsfC3-4	GAGAACACGGGGGACTCTAGAATGAG CGGCGCCGGCGGCATG	GCCCTTGCTCACCATGGATCCGTAGCC GGTGTCCACAGGGA
	TaHsfA2-11	GAGAACACGGGGGACTCTAGAATGGAT CCCTTTCACGGCATTGTGAA	GCCCTTGCTCACCATGGATCCCTGGTA GCTGTGGGGCCGGTTCG
Y2H	TaHsfC3-4	TCAGAGGAGGACCTGCATATGATGAGC GGCGCCGGCGGCATG	TCGACGGATCCCCGGGAATTCTCAGTA GCCGGTGTCCACAGGGA
	TaHsfA2-11	GCCATGGAGGCCAGTGAATTCATGGAT CCCTTTCACGGCATTGTGAA	CAGCTCGAGCTCGATGGATCCTCACTG GTAGCTGTGGGGCCGGTTCG
LCI assays	TaHsfC3-4	ACGGGGGACGAGCTCGGTACCATGAG CGGCGCCGGCGGCATG	CGCGTACGAGATCTGGTCGACGTAGCC GGTGTCCACAGGGA
	TaHsfA2-11	TACGCGTCCCGGGGCGGTACCATGGAT CCCTTTCACGGCATTGTGAA	ACGAAAGCTCTGCAGGTCGACTCACTG GTAGCTGTGGGGCCGGTTCG
